# Knowledge, attitude, and practice toward pulmonary nodules among patients in Northern China: a multicenter cross-sectional study

**DOI:** 10.3389/fpubh.2026.1735755

**Published:** 2026-02-18

**Authors:** Lina Wang, Xuehui Li, Xiaoling Ji, Ying Liu, Yongping Chen, Jian Liu, Yunna Zhou, Xianghong Yu, Honglu Shi, Xueliang Wang

**Affiliations:** 1 Department of Respiratory Medicine, The Fifth People's Hospital of Jinan, Jinan, China; 2 College of Pulmonary and Critical Care Medicine, 8th Medical Center of Chinese PLA General Hospital, Beijing, China; 3 Department of Pulmonary and Critical Care Medicine, 7th Medical Center of Chinese PLA General Hospital, Beijing, China; 4 Department of Respiratory Medicine, Beichen Hospital of Nankai University, Tianjin, China; 5 Department of Respiratory and Critical Care Medicine, Qianxi People's Hospital, Qianxi, China; 6 Health Management Center, Shandong Provincial Hospital Affiliated to Shandong First Medical University, Jinan, China; 7 Department of Medical Imaging and Intervention, Shandong Provincial Hospital Affiliated to Shandong First Medical University, Jinan, China

**Keywords:** cross-sectional study, knowledge attitude practice, Northern China, patient education, pulmonary nodules, structural equation modeling

## Abstract

**Introduction:**

Northern China, characterized by higher rates of lung cancer incidence and pulmonary nodule detection due to elevated air pollution levels and smoking prevalence, necessitates focused assessments. This study aimed to evaluate knowledge, attitudes, and practices (KAP) among pulmonary nodule patients in Northern China.

**Methods:**

This multicenter cross-sectional study was conducted across four hospitals in Northern China between August and December 2024.

**Results:**

A total of 500 patients (95.6% response rate) completed questionnaires. The knowledge, attitudes and practices were 13.82 ± 2.72 (0–18), 31.90 ± 3.44 (9–45), and 34.35 ± 7.22 (13–65), respectively. Spearman analysis revealed significant positive correlations between knowledge-attitude (*r* = 0.271, *p* < 0.001), knowledge-practice (*r* = 0.270, *p* < 0.001), and attitude-practice (*r* = 0.112, *p* = 0.013). Structural equation modeling (SEM) showed that knowledge was significantly associated with attitudes (*β* = 0.553, *p* < 0.001), and attitudes were strongly associated with practices (*β* = 0.865, *p* < 0.001). In addition, a significant indirect association between knowledge and practices through attitudes was observed (*β* = 0.479, *p* < 0.001).

**Conclusion:**

Patients in Northern China exhibit moderate knowledge, neutral attitudes, and suboptimal practices toward pulmonary nodules, with attitudes mediating the knowledge-practice relationship. Targeted interventions should prioritize regional education programs and enhanced clinician-patient communication to improve risk perception and follow-up adherence.

## Introduction

Pulmonary nodules are common findings on chest imaging and are mostly benign, but a subset may represent early-stage lung cancer (1). Approximately 26.3% of these nodules are identified during routine health assessments (2). In China, lung cancer exhibits the highest incidence and mortality rates among all malignant tumors, accounting for nearly 40% of the global burden of new cases and deaths (3). The prevalent application of computed tomography (CT) in clinical evaluations and health screenings has resulted in a notable annual increase in the detection rate of pulmonary nodules (4). Northern China faces a high burden of lung cancer risk factors, including air pollution and smoking, which are associated with increased prevalence of chronic respiratory diseases and abnormal pulmonary findings, as well as disparities in access to specialized care between urban and rural areas. These regional features may influence patients’ understanding of pulmonary nodules and their engagement in recommended follow-up, underscoring the need to examine KAP in this setting (5). Patients’ knowledge, attitudes toward diagnosis and treatment, and health practices concerning pulmonary nodules significantly influence their adherence to early lung cancer screening protocols. This adherence subsequently impacts the rates of early diagnosis and survival among high-risk populations. The Knowledge, Attitude, and Practices (KAP) survey functions as a diagnostic research instrument that elucidates a population’s understanding, beliefs, and behaviors concerning specific health issues (6). This methodology is particularly advantageous in health literacy research, predicated on the notion that knowledge positively influences attitudes, which subsequently shape behaviors (7). In the context of pulmonary nodules, it is essential to examine patients’ Knowledge, Attitudes, and Practices (KAP). Although approximately 96% of pulmonary nodules are ultimately confirmed as benign, previous studies have shown that many patients still experience substantial psychological distress after receiving a pulmonary nodule diagnosis (8).

This so-called “near-cancer” perception, referring to patients’ subjective interpretation of pulmonary nodules as a potential early cancer risk, imposes various burdens on patients, encompassing physical, psychological, and financial challenges (9, 10). Currently, clinicians frequently employ paternalistic or informed decision-making models, which can result in limited information dissemination and diminished satisfaction with the decision-making process (11). Research suggests that the majority of patients with pulmonary nodules express a preference for a more active role in their healthcare decisions; however, they often lack a comprehensive understanding of the natural history of nodules, associated symptoms, and follow-up management options (12–14). Given the distinctive healthcare environment and cultural context in China, coupled with its significant lung cancer burden, targeted research is imperative. Factors such as physician-patient communication patterns, the structure of the healthcare system, and cultural beliefs regarding health decision-making may influence Chinese patients’ understanding and management of pulmonary nodules.

In this context, while a recent study assessed pulmonary nodule–related KAP among the general Chinese adult population (15), evidence remains limited for clinically diagnosed patients, particularly in Northern China, where contextual factors and clinical encounters may shape risk perception and follow-up behaviors. Specifically, Reference 15 reported that although many respondents recognized pulmonary nodules as an important health concern, recommended screening and follow-up behaviors were not consistently adopted, suggesting a potential gap between awareness and action at the population level. This focus on a clinical population, as opposed to general public awareness, provides critical insights into patient-specific needs and behaviors within healthcare contexts. The findings enhance population-level understanding and offer direct implications for improving patient care and management strategies.

## Materials and methods

### Study design and participants

This cross-sectional study involved patients with pulmonary nodules from 4 medical centers in Northern China: the Eighth Medical Center of the Chinese People’s Liberation Army General Hospital, the Shandong Provincial Hospital Affiliated to Shandong First Medical University, the Fifth People’s Hospital of Jinan, Beichen Hospital of Nankai University, conducted between August and December 2024. Inclusion Criteria: (1) Patients diagnosed with pulmonary nodules based on chest CT findings; (2) Patients who consented to complete the questionnaire survey. Exclusion Criteria: (1) Individuals aged younger than 18 or older than 85 years, (2) Responses containing anomalous characters (defined as non-standard symbols, unreadable entries, or response options not included in the predefined questionnaire choices) or logical inconsistencies in the questionnaire, (3) A history of occupational lung diseases, such as pneumoconiosis or silicosis, (4) Previous lung surgery, (5) A diagnosed mental illness, and (6) Intensive care patients. The study was approved by the Medical Ethics Committee of The Fifth People’s Hospital of Jinan (24-5-18). In this study, screening and follow-up were based on low-dose computed tomography (LDCT) detection and risk-stratified surveillance intervals for pulmonary nodules, in accordance with the Chinese Expert Consensus on the Diagnosis and Treatment of Pulmonary Nodules. All participants were informed about the study protocol and provided written informed consent prior to participation. All methods were performed in accordance with relevant institutional and national research guidelines. All procedures were performed in accordance with the ethical standards laid down in the 1964 Declaration of Helsinki and its later amendments.

### Questionnaire introduction

The questionnaire utilized in this study was meticulously developed in accordance with the “Chinese Expert Consensus on Diagnosis and Treatment of Pulmonary Nodules” (16) and pertinent literature (17). An initial draft was subjected to a pilot test, which yielded 30 valid responses, achieving a response rate of 100%. The overall Cronbach’s *α* coefficient was calculated to be 0.938, indicating a high level of internal consistency. Content validity was assessed by an expert panel consisting of two senior respiratory physicians and one epidemiology specialist, who reviewed the questionnaire items for clinical relevance, clarity, and domain coverage. Minor wording revisions were made based on their feedback. Construct validity was evaluated using confirmatory factor analysis (CFA), which supported the three-factor structure (knowledge, attitude, and practice). The model demonstrated acceptable fit indices (RMSEA = 0.064, SRMR = 0.073, TLI = 0.803, and CFI = 0.831), indicating an adequate measurement model fit (Supplementary Table 1; Supplementary Figure 1). Specifically, the knowledge dimension exhibited a coefficient of 0.848, the attitude dimension 0.847, and the practice dimension 0.932. The final iteration of the questionnaire, presented in Chinese, is systematically organized into four sections. These sections gather demographic information, including age, gender, body mass index (BMI), place of residence, educational attainment, ethnicity, occupational status, income, marital status, smoking and drinking behaviors, medical insurance coverage, duration since diagnosis, exposure history to dust, asbestos, and oil fumes, medical history, family history of lung cancer, regularity of physical examinations, fertility history, abortion history, and progesterone use. Furthermore, the questionnaire assesses knowledge, attitudes, and practices. The knowledge section comprises 9 items, each scored on a scale where participants receive 2 points for “very familiar,” 1 point for “heard of,” and 0 points for “unclear,” resulting in a total score range of 0 to 18 points. The attitude section similarly contains 9 items, employing a five-point Likert scale ranging from “strongly agree” to “strongly disagree,” with scores varying from 5 to 1, yielding a total score range of 9 to 45 points. The practice dimension encompasses 13 items, scored from 5 to 1 based on responses ranging from “always” to “never,” resulting in a score range of 13 to 65 points. Scores exceeding 75% of the maximum possible in each dimension indicate adequate knowledge, a positive attitude, and proactive practices. Scores falling between 50 and 75% of the maximum reflect moderate knowledge, a neutral attitude, and moderate practices, Scores below 50% of the maximum possible indicate insufficient knowledge, negative attitudes, and poor practices (18).

### Questionnaire distribution and quality control

A convenience sampling survey methodology was employed, involving the distribution of offline questionnaires across four hospitals located in North China. To assist participants encountering difficulties during the survey, designated members of the research team were available to address their concerns. For the purposes of quality control, the research team comprised ten members who underwent standardized training prior to the commencement of the project. This training emphasized the significance of the questionnaire content, the specific components evaluated in each question, strategies for addressing participants’ inquiries, and effective communication techniques. During the offline collection of questionnaires, team members actively supported participants by promptly responding to inquiries and ensuring a clear understanding of the questions posed. In addition, during routine outpatient visits, clinicians provided standardized verbal explanations regarding pulmonary nodule screening procedures, follow-up recommendations, and potential management options. For participants with limited literacy, trained staff offered face-to-face assistance and oral explanations to facilitate comprehension before questionnaire completion.

### Sample size

Sample size was calculated using the formula for a cross-sectional study (19): α =0.05, $n={\left(\frac{Z_{1-\alpha /2}}{\delta }\right)}^{2}\times p\times (1-p)$ where $Z_{1-\alpha /2}$ =1.96 when α =0.05, the assumed degree of variability of p =0.5 maximizes the required sample size, and δ is admissible error (which was 5% here). The theoretical sample size was 480 which includes an extra 20% to allow for subjects lost during the study.

### Statistical analysis

Statistical analysis was conducted utilizing SPSS version 26.0 (IBM Corp., Armonk, N. Y., USA) and AMOS version 21.0 (Amos Development Corporation, Chicago, IL). Continuous variables were assessed for normality and reported as means with standard deviations (SD) or medians with interquartile ranges (IQR), contingent upon their distribution characteristics. Categorical variables were expressed as frequencies and percentages (n, %). Continuous variables exhibiting a normal distribution were compared using independent sample t-tests or analysis of variance (ANOVA), whereas those with skewed distributions were analyzed employing the Wilcoxon-Mann–Whitney test or Kruskal-Wallis ANOVA. Correlations were evaluated through Pearson or Spearman correlation coefficients (20), dependent on the distribution of the data. Univariate and multivariate regression analyses were conducted to explore associations between demographic variables and levels of knowledge, attitudes, and proactive practices. Furthermore, a structural equation modeling (SEM) analysis was undertaken to investigate the interrelationships among knowledge, attitudes, and practices (KAP). The hypotheses tested within the SEM framework included: (1) knowledge is associated with attitude, (2) attitude is associated with practice, and (3) knowledge is associated with practice both directly and indirectly through attitude. All statistical analyses employed a two-sided test, with *p*-values < 0.05 were considered statistically significant. Model fit was evaluated using several commonly recommended indices: the Root Mean Square Error of Approximation (RMSEA, acceptable if <0.08), the Standardized Root Mean Square Residual (SRMR, acceptable if <0.08), the Tucker–Lewis Index (TLI, acceptable if >0.80), and the Comparative Fit Index (CFI, acceptable if >0.80). These indices were applied to assess the adequacy of the structural equation model. In exploratory cross-sectional behavioral models, incremental fit indices slightly below conventional thresholds may still be interpreted as indicating marginal or acceptable model fit when considered together with other indices such as RMSEA and SRMR; however, such interpretations should be made with caution.

## Results

### Demographic characteristics

**Figure 1:**
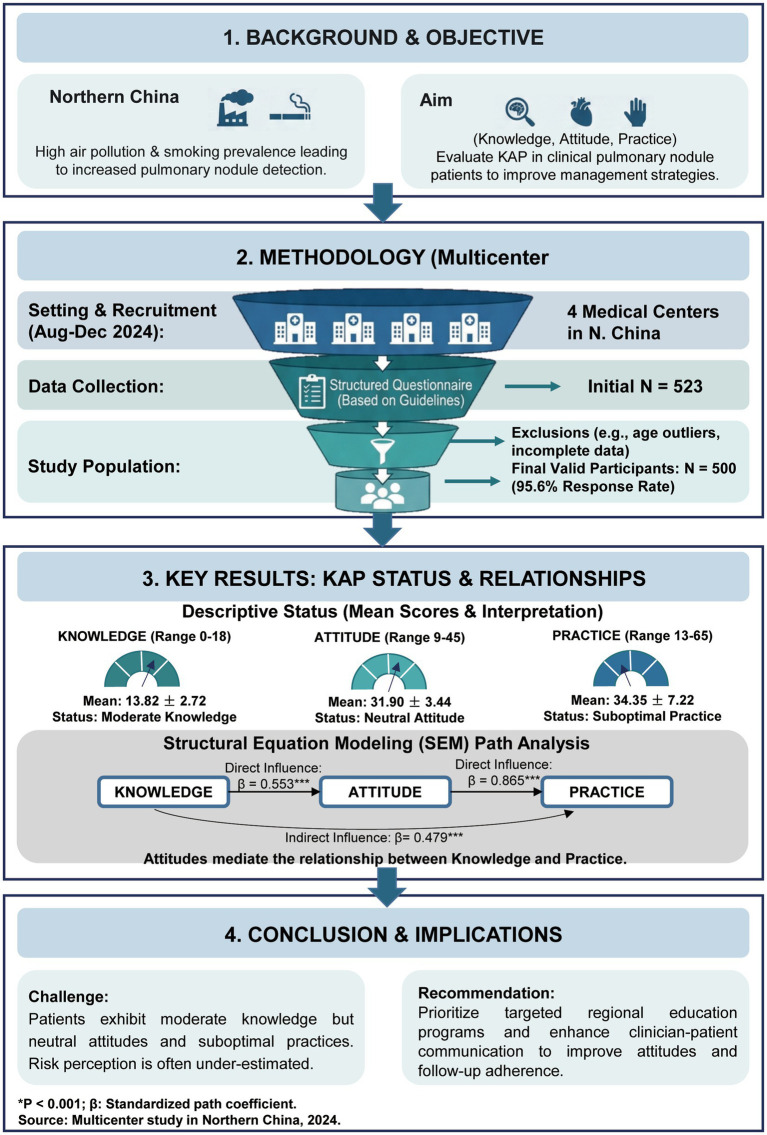
Study flowchart.

A total of 523 questionnaires were collected, of which 23 were excluded due to invalid responses. Specifically, 18 cases were excluded due to the presence of outliers or response options not included in the questionnaire within the baseline section. Additionally, 5 cases were eliminated for failing to respond in accordance with the available options. Consequently, 500 valid patient questionnaires were included in the analysis, resulting in a valid response rate of 95.60% (Figure 1). The questionnaire demonstrated acceptable internal consistency and an adequate sample size, evidenced by a Cronbach’s alpha of 0.8468 and a Kaiser-Meyer-Olkin (KMO) value of 0.8234.

Among the valid responses, 175 participants (35.0%) were aged between 50 and 64 years, 252 (50.4%) identified as female, and 362 (72.4%) resided in urban areas. Additionally, 191 respondents (38.2%) held an associate or bachelor’s degree, 199 (39.8%) were employed, and 94 (18.8%) were current smokers. Furthermore, 302 participants (60.4%) had been diagnosed with pulmonary nodules for less than one year, 112 (22.4%) had a history of long-term exposure to dust, asbestos, or cooking oil fumes, 129 (25.8%) had experienced related diseases, and 264 (52.8%) underwent regular physical examinations (Table 1).

**Table 1:** Demographic characteristics and knowledge, attitude, and practice scores.

*N* = 500	*N* (%)	Knowledge	*p*	Attitude	*p*	Practice	*p*
		Mean (SD)		Mean (SD)		Mean (SD)	
Total score	500 (100.0)	13.82 (2.72)		31.90 (3.44)		34.35 (7.22)	
Age			**<0.001**		0.572		**<0.001**
18–34	88 (17.6)	14.55 (2.43)		32.45 (3.92)		36.26 (8.09)	
35–49	130 (26.0)	14.62 (2.58)		31.94 (3.19)		36.09 (7.46)	
50–64	175 (35.0)	13.11 (2.73)		31.60 (3.51)		33.42 (6.55)	
≥ 65	107 (21.4)	13.41 (2.74)		31.89 (3.16)		32.19 (6.36)	
Gender			0.575		0.463		0.765
Male	248 (49.6)	13.74 (2.70)		32.08 (3.39)		34.48 (7.45)	
Female	252 (50.4)	13.90 (2.75)		31.72 (3.47)		34.23 (6.99)	
BMI			0.178		0.109		0.369
< 18.50	20 (4.0)	13.60 (3.45)		33.30 (4.17)		36.30 (10.29)	
18.50–23.99	237 (47.4)	13.87 (2.63)		31.63 (3.29)		34.49 (6.98)	
24.00–27.99	189 (37.8)	14.01 (2.74)		31.94 (3.41)		34.40 (7.38)	
≥ 28.00	54 (10.8)	13.06 (2.75)		32.43 (3.75)		32.89 (6.23)	
Residence			**0.001**		0.452		0.549
Urban	362 (72.4)	14.06 (2.72)		31.80 (3.45)		34.29 (6.83)	
Rural	138 (27.6)	13.20 (2.65)		32.15 (3.40)		34.51 (8.18)	
Education			**<0.001**		0.364		**<0.001**
Primary school or below	55 (11.0)	12.15 (2.96)		31.20 (3.40)		31.36 (7.92)	
Junior high school	113 (22.6)	12.88 (2.66)		31.68 (2.89)		31.72 (5.28)	
High school/technical school	110 (22.0)	13.95 (2.50)		31.85 (3.41)		34.41 (7.02)	
Associate /bachelor’s degree	191 (38.2)	14.72 (2.43)		32.20 (3.54)		36.58 (7.21)	
Master’s degree or above	31 (6.2)	14.23 (2.69)		32.26 (4.58)		35.35 (8.13)	
Ethnicity			0.314		**0.049**		0.479
Han	474 (94.8)	13.85 (2.71)		31.97 (3.46)		34.32 (7.23)	
Minority	26 (5.2)	13.23 (2.94)		30.58 (2.69)		34.96 (7.10)	
Employment status			**<0.001**		0.075		**<0.001**
Employed	199 (39.8)	14.59 (2.47)		32.06 (3.56)		36.32 (7.42)	
Unemployed	36 (7.2)	12.89 (2.90)		32.39 (3.05)		32.69 (5.40)	
Retired	137 (27.4)	13.65 (2.80)		32.04 (3.45)		32.95 (6.64)	
Self-employed	31 (6.2)	13.58 (2.58)		32.39 (4.37)		35.84 (8.82)	
Other	97 (19.4)	12.91 (2.69)		31.03 (2.82)		32.44 (6.59)	
Monthly income			**<0.001**		**0.001**		**<0.001**
<2000	89 (17.8)	12.06 (2.64)		30.73 (2.95)		31.42 (6.26)	
2000–5,000	147 (29.4)	13.50 (2.70)		31.66 (3.30)		32.74 (6.61)	
5,000–10,000	174 (34.8)	14.58 (2.38)		32.56 (3.60)		35.57 (6.88)	
>10,000	90 (18.0)	14.63 (2.56)		32.18 (3.47)		37.53 (7.99)	
Marital status			0.441		0.348		0.199
Married	425 (85.0)	13.87 (2.68)		31.96 (3.47)		34.53 (7.14)	
Single/divorced/widowed (single)	75 (15.0)	13.53 (2.96)		31.55 (3.24)		33.36 (7.60)	
Smoking habit			0.060		0.382		0.593
Never	312 (62.4)	14.05 (2.69)		32.08 (3.65)		34.19 (7.26)	
Used to smoke	94 (18.8)	13.56 (2.69)		31.65 (3.12)		34.57 (6.94)	
Currently smoking	94 (18.8)	13.33 (2.81)		31.55 (2.97)		34.68 (7.40)	
Alcohol habit			0.618		0.676		**0.027**
Never	281 (56.2)	13.88 (2.70)		31.73 (3.42)		33.67 (6.77)	
Used to drink	95 (19.0)	13.87 (2.86)		32.15 (3.53)		34.43 (7.60)	
Currently drinking	124 (24.8)	13.65 (2.69)		32.10 (3.40)		35.83 (7.72)	
Medical insurance (multiple choice)			**<0.001**		0.264		**0.009**
Urban employee basic medical insurance	335 (67.0)	14.26 (2.62)		32.12 (3.54)		34.81 (7.15)	
Urban resident basic medical insurance	147 (29.4)	12.88 (2.72)		31.44 (3.26)		33.17 (7.31)	
Other	18 (3.6)	13.39 (2.79)		31.67 (2.47)		35.44 (7.00)	
Duration of pulmonary nodules			**<0.001**		**0.005**		0.100
Less than 1 year	302 (60.4)	13.40 (2.73)		31.64 (3.50)		33.99 (7.27)	
1–2 years	108 (21.6)	14.19 (2.55)		31.81 (3.25)		34.72 (7.86)	
More than 2 years	90 (18.0)	14.78 (2.63)		32.87 (3.29)		35.12 (6.16)	
Long-term exposure to dust, asbestos, cooking oil fumes, etc.			0.216		0.153		0.119
No	388 (77.6)	13.90 (2.69)		32.04 (3.42)		33.97 (6.91)	
Yes	112 (22.4)	13.54 (2.83)		31.42 (3.47)		35.67 (8.08)	
Diseases (multiple choice)			0.169		0.071		0.601
No	371 (74.2)	13.92 (2.70)		32.05 (3.38)		34.30 (7.18)	
Yes	129 (25.8)	13.53 (2.78)		31.46 (3.56)		34.50 (7.34)	
Chronic obstructive pulmonary disease	28 (5.6)	13.57 (2.94)		32.11 (3.05)		33.79 (6.87)	
Tuberculosis	15 (3.0)	12.33 (2.72)		29.80 (3.14)		35.27 (9.48)	
Pulmonary fibrosis	7 (1.4)	14.14 (3.08)		31.29 (2.06)		36.43 (2.76)	
Chronic bronchitis	70 (14.0)	13.81 (2.63)		31.77 (3.70)		34.97 (7.64)	
Malignant tumor	9 (1.8)	12.67 (3.12)		29.89 (4.70)		30.22 (2.95)	
Family history of lung cancer			0.714		0.731		**0.005**
No	460 (92.0)	13.80 (2.73)		31.88 (3.47)		34.17 (7.31)	
Yes	40 (8.0)	14.03 (2.71)		32.12 (3.00)		36.48 (5.74)	
Regular physical examinations			**<0.001**		**<0.001**		**0.001**
No	236 (47.2)	13.24 (2.81)		31.09 (3.07)		33.25 (6.71)	
Yes	264 (52.8)	14.34 (2.53)		32.62 (3.58)		35.33 (7.52)	

*p* < 0.05.

### Knowledge, attitude, and practice dimensions

The mean knowledge, attitude, and practice were 13.82 ± 2.72 (possible range: 0–18), 31.90 ± 3.44 (possible range: 9–45), and 34.35 ± 7.22 (possible range: 13–65), respectively. The results showed that 299 participants (59.8%) had adequate knowledge, 269 participants (53.8%) had positive attitudes, and 231 participants (46.2%) demonstrated proactive practices. Knowledge scores exhibited significant variation based on age (*p* < 0.001), residence (*p* = 0.001), education (*p* < 0.001), employment status (*p* < 0.001), monthly income (*p* < 0.001), medical insurance (*p* < 0.001), duration of pulmonary nodules (*p* < 0.001), and regular physical examinations (p < 0.001). Attitude scores varied significantly by ethnicity (*p* = 0.049), monthly income (*p* = 0.001), duration of pulmonary nodules (*p* = 0.005), and regular physical examinations (*p* < 0.001). Practice scores varied significantly by age (*p* < 0.001), education (*p* < 0.001), employment status (*p* < 0.001), monthly income (*p* < 0.001), alcohol habit (*p* = 0.027), medical insurance (*p* = 0.009), family history of lung cancer (*p* = 0.005), and regular physical examinations (*p* = 0.001) (Table 1).

The distribution of knowledge dimensions revealed that the three questions with the highest percentage of participants selecting the “Unclear” option were: “The probability of malignancy varies with the density of pulmonary nodules. Solid or mixed-density nodules have a higher likelihood of malignancy” (K5) at 52.8%; “Pulmonary nodules are defined as round or irregular lesions in the lung with a diameter of less than or equal to 3 cm” (K1) at 52.2%; and “Pulmonary nodules may be caused by inflammation or autoimmune diseases” (K2) at 42.8% (Supplementary Table 2).

In the dimension of attitudes, the responses indicated that 14.8% of participants strongly agreed and 22.6% agreed that they often experience anxiety following a diagnosis of pulmonary nodules (A7). Furthermore, 14% strongly agreed and 23.2% agreed that they harbor concerns regarding exposure to X-ray radiation during screening or follow-up procedures (A8). Additionally, 10.8% strongly agreed and 12% agreed that most pulmonary nodules are benign and do not necessitate follow-up or re-examination (A3) (Supplementary Table 3).

With respect to the practice dimension, when evaluating exposure to risk factors in daily life (P2), 12% reported consistently being exposed to asbestos, dust, and similar hazards, while 10.8% reported regular exposure to air pollution. When queried about preferred further examinations or treatments, 37.4% indicated they would consistently consider surgical resection (P5.5), whereas over 44.4% never contemplated alternative options (P5.1-P5.4). Moreover, in the context of significant psychological stress, 9.2% reported always and 25.2% often requesting treatment for the lesion, even when not recommended by their healthcare provider (P6) (Supplementary Table 4).

### Correlation analysis

Spearman correlation analysis revealed significant positive relationships between knowledge and attitude (*r* = 0.271, *p* < 0.001), between attitude and practice (*r* = 0.112, *p* = 0.013) and between knowledge and practice (*r* = 0.270, *p* < 0.001) (Table 2).

**Table 2:** Spearman correlation analysis.

Spearman correlation analysis	Knowledge dimension	Attitude dimension	Practice dimension
Knowledge dimension	1.000		
Attitude dimension	0.271 (*p* < 0.001)	1.000	
Practice dimension	0.270 (*p* < 0.001)	0.112 (*p* = 0.013)	1.000

### Univariable and multivariable logistic regression analysis

The median of the knowledge, attitude, and practice scores were used as the cut-off value for each dimension to divided the groups, and the number of participants above the cut-off value were 299 (59.8%), 269 (53.8%), and 231 (46.2%), respectively. The multivariable logistic regression analysis revealed that the influencing factor for practice scores were only the knowledge scores (OR = 1.083, 95% CI: 1.042, 1.127, *p* < 0.001) and a family history of lung cancer (OR = 2.571, 95% CI: 1.154, 5.729, *p* = 0.021) (Table 3).

**Table 3:** Univariable and multivariable logistic regression.

Practice	Univariate analysis	*p*	Multivariate analysis	*p*
	OR (95%CI)		OR (95%CI)	
Knowledge	1.174 (1.097, 1.256)	<0.001	1.105 (1.021, 1.195)	0.013
Attitude	1.080 (1.024, 1.139)	0.005	1.042 (0.981, 1.106)	0.182
Age				
18–34				
35–49	1.434 (0.814, 2.525)	0.212		
50–64	0.683 (0.404, 1.146)	0.151		
≥ 65	0.648 (0.365, 1.144)	0.137		
Gender				
Male				
Female	0.996 (0.699, 1.419)	0.980		
BMI				
<18.50				
18.50–23.99	0.913 (0.346, 2.291)	0.849		
24.00–27.99	0.970 (0.365, 2.456)	0.949		
> = 28.00	0.495 (0.169, 1.389)	0.187		
Residence				
Urban				
Rural	0.837 (0.564, 1.243)	0.376		
Education				
Primary school or below				
Junior high school	1.380 (0.719, 2.691)	0.337	1.221 (0.594, 2.511)	0.587
High school/technical school	2.253 (1.169, 4.421)	0.016	1.791 (0.809, 3.963)	0.151
Associate /bachelor’s degree	3.290 (1.780, 6.205)	<0.001	1.897 (0.790, 4.550)	0.152
Master’s degree or above	2.564 (1.050, 6.474)	0.041	1.164 (0.365, 3.708)	0.798
Ethnicity				
Han				
Minority	1.230 (0.554, 2.859)	0.617		
Employment status				
Employed				
Unemployed	0.695 (0.338, 1.456)	0.325	1.043 (0.408, 2.666)	0.930
Retired	0.504 (0.321, 0.786)	0.003	0.706 (0.395, 1.260)	0.239
Self-employed	0.529 (0.246, 1.145)	0.103	0.584 (0.233, 1.464)	0.251
Other	0.429 (0.260, 0.704)	0.001	0.710 (0.335, 1.509)	0.374
Monthly income				
<2000				
2000–5,000	1.349 (0.795, 2.304)	0.269	0.996 (0.523, 1.896)	0.990
5,000–10,000	2.476 (1.474, 4.201)	0.001	1.361 (0.689, 2.692)	0.375
>10,000	3.460 (1.876, 6.512)	<0.001	1.994 (0.889, 4.471)	0.094
Marital status				
Married				
Single/divorced/widowed (single)	0.747 (0.456, 1.224)	0.246		
Smoking habit				
Never				
Used to smoke	1.483 (0.928, 2.397)	0.103		
Currently smoking	1.483 (0.928, 2.397)	0.103		
Alcohol habit				
Never				
Used to drink	1.450 (0.906, 2.342)	0.125	1.377 (0.826, 2.295)	0.220
Currently drinking	1.681 (1.091, 2.614)	0.020	1.591 (0.993, 2.547)	0.053
Medical insurance (multiple choice)				
Urban employee basic medical insurance				
Urban resident basic medical insurance	0.648 (0.438, 0.957)	0.029	1.244 (0.668, 2.319)	0.492
Other	1.350 (0.511, 3.958)	0.558	1.425 (0.465, 4.362)	0.535
Duration of pulmonary nodules				
Less than 1 year				
1–2 years	1.196 (0.769, 1.870)	0.428	0.938 (0.578, 1.523)	0.795
More than 2 years	1.965 (1.202, 3.278)	0.008	1.595 (0.908, 2.803)	0.104
Long-term exposure to dust, asbestos, cooking oil fumes, etc.				
No				
Yes	1.291 (0.843, 1.996)	0.244		
Diseases (multiple choice)				
No				
Yes	1.279 (0.852, 1.934)	0.238		
Family history of lung cancer				
No				
Yes	2.818 (1.365, 6.411)	0.008	2.571 (1.154, 5.729)	0.021
Regular physical examinations				
No				
Yes	1.436 (1.007, 2.051)	0.046	0.828 (0.526, 1.302)	0.413

The median of the knowledge, attitude, and practice scores were used as the cut-off value for each dimension. The number of participants above the cut-off value were 299 (59.8%), 269 (53.8%), and 231 (46.2%), respectively.

### SEM analysis

**Figure 2:**
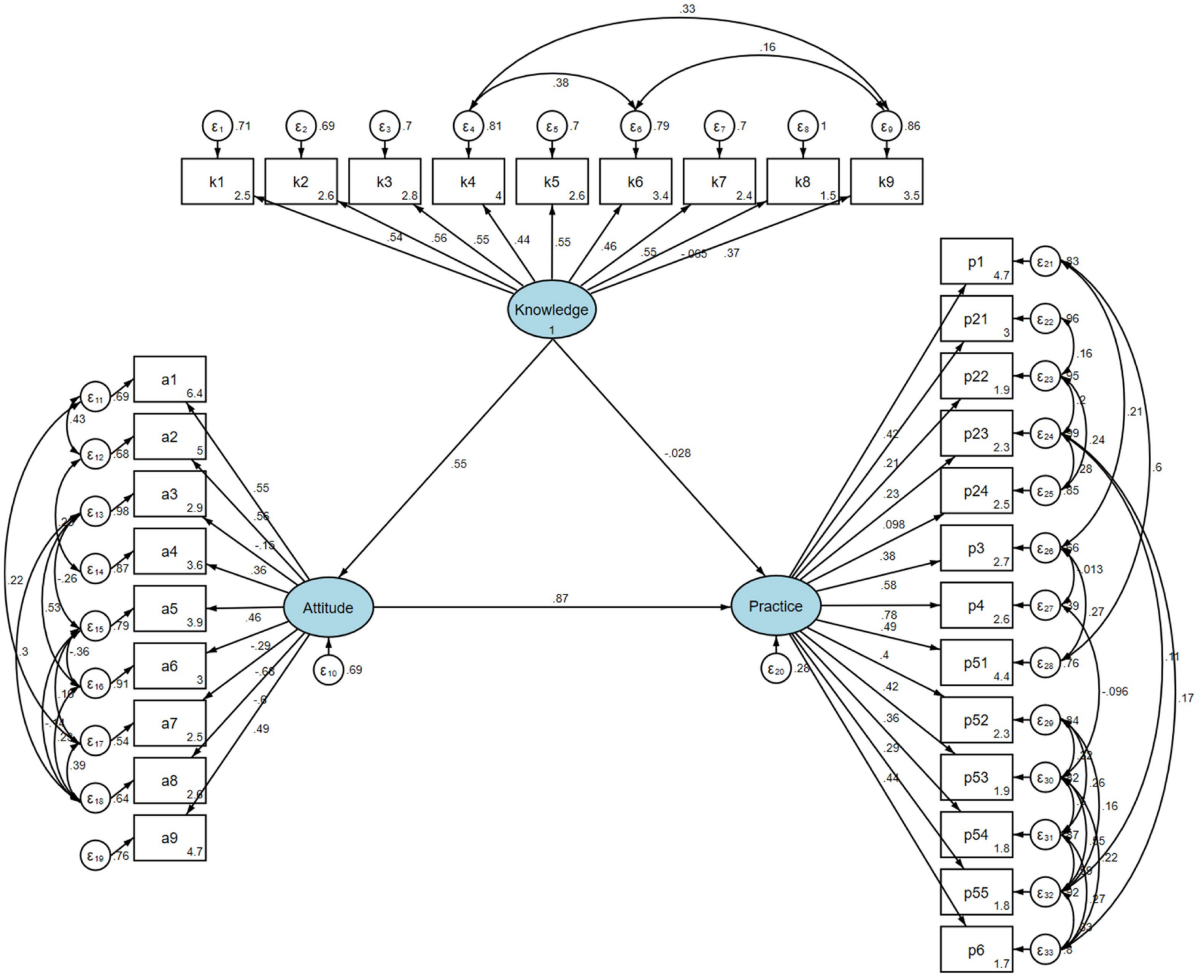
Structural equation modeling (SEM) of the relationships among knowledge, attitude, and practice. Ellipses represent latent variables, rectangles indicate observed questionnaire items, and single-headed arrows denote standardized path coefficients. Curved double-headed arrows indicate correlated error terms. *ε* represents measurement error.

SEM found that knowledge had direct association with attitude (*β* = 0.553, 95% CI: 0.444, 0.662, *p* < 0.001), attitude had direct association with practice (*β* = 0.865, 95% CI: 0.741, 0.989, *p* < 0.001), Knowledge had indirect association with practice through attitude (*β* = 0.479, 95% CI: 0.343, 0.615, *p* < 0.001) (Table 4). The SEM structure is presented in Figure 2.

**Table 4:** Mediation analysis.

Model paths	Total effects		Direct Effect		Indirect effect	
	β (95%CI)	*p*	β (95%CI)	*p*	β (95%CI)	*p*
Attitude						
Knowledge	0.553 (0.444, 0.662)	<0.001	0.553 (0.444, 0.662)	<0.001		
Practice						
Knowledge	0.451 (0.339, 0.563)	<0.001	−0.028 (−0.178, 0.123)	0.718	0.479 (0.343, 0.615)	<0.001
Attitude	0.865 (0.741, 0.989)	<0.001	0.865 (0.741, 0.989)	<0.001		

## Discussion

This study demonstrated that patients with pulmonary nodules in Northern China exhibited moderate knowledge, neutral attitudes, and suboptimal practices. Structural equation modeling indicated that knowledge was significantly associated with attitude and indirectly associated with practice through attitudes, highlighting the potential mediating role of attitudes in health-related behaviors. In addition, a family history of lung cancer significantly increased the likelihood of proactive practices. These findings highlight the importance of improving knowledge and risk communication to promote more positive attitudes and better adherence to recommended management strategies.

Compared to the general population study by Cheng et al. (15), our clinical population in Northern China showed different KAP patterns. While the general population demonstrated higher knowledge scores (mean 14.2 vs. 13.82 in our study), our clinical patients showed more positive attitudes toward pulmonary nodule management, likely due to their direct experience with the condition. Recent research involving 1,209 Chinese adults has revealed a paradox in the management of pulmonary nodules. While 62.3% acknowledged these nodules as significant health threats, only 38.7% engaged in recommended screening practices. This discrepancy is particularly pronounced among individuals with higher educational attainment and socioeconomic status (15). Additionally, the population’s overall neutral attitudes, reflected by a mean score of 3.2 out of 5, suggest a systemic underestimation of the long-term risks associated with pulmonary nodules. This lack of awareness may be exacerbated by insufficient risk communication in clinical settings, despite an increasing general awareness of health issues. Structural equation modeling suggests that knowledge is significantly associated with attitudes, and attitudes are strongly associated with practice patterns. These findings align with existing literature suggesting that interventions aimed at enhancing knowledge can positively affect patient attitudes, ultimately promoting adherence to recommended healthcare practices. Mediation analysis suggests that the association between knowledge and practice may be partially explained by attitudes, emphasizing the importance of considering cognitive and emotional factors in patient decision-making. In China, factors such as healthcare accessibility and economic considerations significantly influence patient engagement with follow-up care and management strategies. Comparable studies have shown that while knowledge is crucial for behavior change, attitude often serves as the key mediator from awareness to action (21).

The apparent discrepancy between the logistic regression and SEM findings reflects fundamental differences in the analytical objectives and statistical frameworks of these two approaches. Logistic regression evaluates the independent association between predictors and the outcome while adjusting for covariates, and in this model knowledge remained a significant predictor of practice, whereas attitude did not retain independent statistical significance. In contrast, SEM simultaneously models multiple interrelated pathways and emphasizes latent structural relationships rather than isolated predictors. In the SEM framework, attitude demonstrated a strong association with practice, whereas the direct association between knowledge and practice was not statistically significant, suggesting that the influence of knowledge on practice may be primarily transmitted through attitudinal pathways rather than acting independently. Therefore, these models address complementary research questions: regression analysis identifies independent predictors of practice, whereas SEM explores the underlying relational structure among knowledge, attitudes, and practices. Taken together, the findings support an integrated interpretation in which knowledge contributes to practice mainly through its association with attitudes, while also retaining an independent predictive role in traditional regression models.

Multivariate analysis identified several sociodemographic factors that significantly influence knowledge levels. Patients with higher educational attainment exhibited superior understanding, consistent with health literacy research that emphasizes the role of formal education in shaping disease awareness (22). Additionally, higher income levels correlated with enhanced knowledge, likely due to increased access to healthcare resources and information. Empirical studies have indicated that financial stability fosters greater engagement with healthcare services, thereby improving disease awareness and self-management practices (23, 24). Moreover, a longer duration of illness was associated with higher knowledge scores, suggesting that frequent medical consultations provide more opportunities for patients to acquire relevant information. Prior research indicates that regular interactions with healthcare professionals significantly enhance patient knowledge and self-efficacy in disease management (25).

Attitudes were also influenced by various sociodemographic factors. Higher knowledge levels were significantly correlated with more positive attitudes, a trend well-documented in health behavior literature (26). Employment status played a crucial role; unemployed or retired individuals exhibited more favorable attitudes toward managing pulmonary nodules. This observation aligns with previous research indicating that individuals with more free time tend to engage more actively in their healthcare, resulting in a greater willingness to adhere to medical recommendations (27, 28). Furthermore, regular participation in physical examinations was associated with more positive attitudes, underscoring the importance of preventive healthcare engagement in shaping patient perspectives (29). These findings suggest that incorporating targeted educational initiatives during routine health check-ups could effectively enhance patient attitudes toward managing pulmonary nodules.

In contrast, practice scores exhibited only a moderate correlation with knowledge and attitudes. This finding suggests that while information and perceptions are significant, other factors also influence patient behaviors. A notable predictor of proactive health behaviors identified in the study was a family history of lung cancer, consistent with research indicating that perceived personal risk drives health-related actions (30). Conversely, financial and employment status did not significantly impact practice scores, implying that although economic constraints may hinder access to healthcare, other psychological and structural barriers also contribute to behavioral adherence (31).

To address these gaps, an integrated approach is necessary, combining systemic healthcare improvements with targeted educational initiatives. Enhancing physician-patient communication is essential, as effective information delivery significantly improves patient comprehension and adherence (32). Healthcare institutions should consider implementing structured educational programs that provide clear and accessible information on managing pulmonary nodules, including risk stratification and follow-up guidelines. Additionally, digital health tools, such as mobile applications and online resources, could increase patient engagement and facilitate ongoing education (33).

Educational interventions should specifically target identified knowledge gaps and address prevalent misconceptions. The use of visual aids, simplified educational materials, and structured counseling sessions can assist patients in achieving a more comprehensive understanding of their health conditions. Community outreach programs, particularly in rural areas with lower health literacy scores, can enhance health literacy and encourage proactive engagement with healthcare services. Integrating patient education into primary care settings and fostering shared decision-making between healthcare providers and patients could further improve attitudes and adherence to recommended behaviors. Research indicates that patient-centered educational interventions emphasizing interactive learning and tailored messaging are more effective in promoting sustained health behavior change (34).

This study has several limitations. First, its cross-sectional design limits the ability to establish causal relationships between demographic factors and KAP components, thereby restricting inferences about the directionality of the observed associations. Second, reliance on self-reported data may introduce recall or social desirability bias, potentially affecting the accuracy of the findings. Lastly, the sample was drawn from healthcare institutions in Northern and Eastern China, which may limit the generalizability of the results to other regions or populations with differing sociodemographic characteristics. In addition, the cross-sectional design precludes causal inference in the SEM analysis, and the relatively modest model fit indices suggest that the structural relationships should be interpreted as associative rather than causal.

In conclusion, this multicenter study found that patients with pulmonary nodules in Northern China generally demonstrated moderate knowledge, neutral attitudes, and suboptimal practices. Knowledge influenced practices indirectly through attitudes, while a family history of lung cancer further promoted proactive behaviors. These findings highlight the importance of strengthening patient education, improving physician–patient communication, and addressing systemic barriers to follow-up care. Future longitudinal studies are warranted to clarify the evolution of KAP over time and to evaluate innovative approaches, including digital tools, for personalized management of pulmonary nodules.

## Data Availability

The original contributions presented in the study are included in the article/Supplementary material, further inquiries can be directed to the corresponding authors.
