# Supplementary material for: Knowledge, attitude, and practice toward pulmonary nodules among patients in Northern China: a multicenter cross-sectional study
Source: Front Public Health. 2026 Feb 18;14:1735755. doi: 10.3389/fpubh.2026.1735755 (PMC12957129; doi:10.3389/fpubh.2026.1735755)
Supplement: Supplementary file 1 [file Data_Sheet_1.docx]

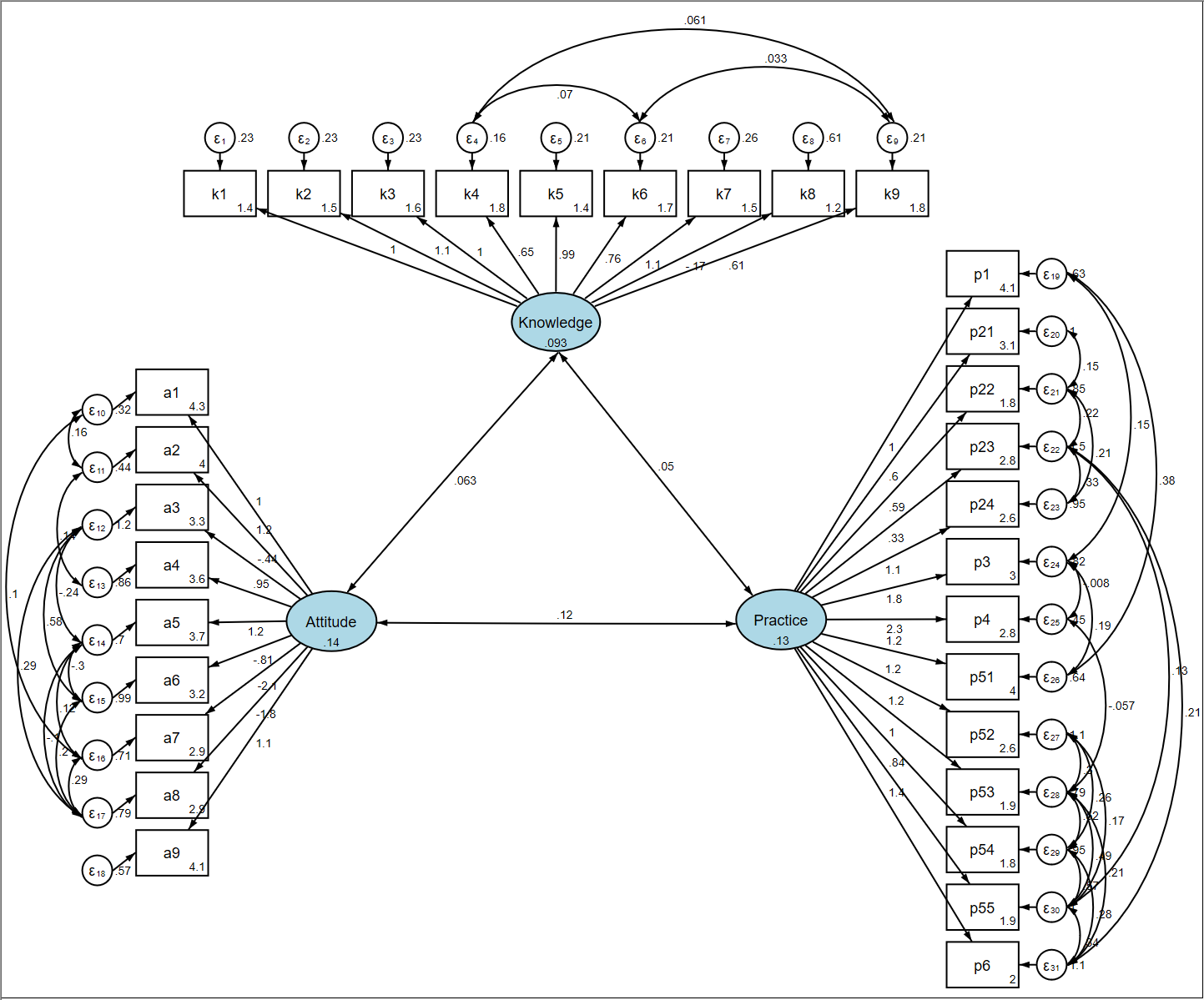


**Supplementary Figure 1. CFA path**

**Supplementary Table 1. CFA fit**

| **Indicators** | **Reference** | **Results** |
| --- | --- | --- |
| RMSEA | <0.08 | 0.064 |
| SRMR | <0.08 | 0.073 |
| TLI | >0.80 | 0.803 |
| CFI | >0.80 | 0.831 |

**Supplementary Table 2. Distribution of knowledge dimension responses.**

| **Knowledge** | True | False | Not sure |
| --- | --- | --- | --- |
| 1.Pulmonary nodules are defined as round or irregular lesions in the lung with a diameter of less than or equal to 3 cm. | 219 (43.8%) | 20 (4%) | 261 (52.2%) |
| 2.Pulmonary nodules may be caused by inflammation or autoimmune diseases. | 266 (53.2%) | 20 (4%) | 214 (42.8%) |
| 3.Pulmonary nodules are not equivalent to lung cancer, and most pulmonary nodules are benign. | 337 (67.4%) | 24 (4.8%) | 139 (27.8%) |
| 4.Physical examinations are an important way to detect early-stage lung cancer. Early detection, diagnosis, and treatment can improve lung cancer survival rates. | 403 (80.6%) | 9 (1.8%) | 88 (17.6%) |
| 5.The probability of malignancy varies with the density of pulmonary nodules. Solid or mixed-density nodules have a higher likelihood of malignancy. | 221 (44.2%) | 15 (3%) | 264 (52.8%) |
| 6.Chest CT is the most effective method for detecting pulmonary nodules. | 372 (74.4%) | 14 (2.8%) | 114 (22.8%) |
| 7.Pulmonary nodules without risk factors do not require follow-up. For patients with lung cancer risk factors, re-evaluation is recommended 3-6 months later. If there is no change in the nodule, routine annual checks can be adopted. | 270 (54%) | 31 (6.2%) | 199 (39.8%) |
| 8.Newly detected small pulmonary nodules (0.5 cm-1.0 cm) can be ignored. | 119 (23.8%) | 199 (39.8%) | 182 (36.4%) |
| 9.Early intervention is crucial for pulmonary nodules assessed by modern medical guidelines as requiring regular follow-up. | 391 (78.2%) | 15 (3%) | 94 (18.8%) |

**Supplementary Table 3. Distribution of attitude dimension responses.**

| **Attitude** | **Strongly agree** | **Agree** | **Neutral** | **Disagree** | **Strongly disagree** |
| --- | --- | --- | --- | --- | --- |
| 1.I believe pulmonary nodules should be taken seriously. | 219 (43.8%) | 242 (48.4%) | 30 (6%) | 8 (1.6%) | 1 (0.2%) |
| 2.I believe pulmonary nodules increase the risk of developing lung cancer. | 144 (28.8%) | 234 (46.8%) | 104 (20.8%) | 17 (3.4%) | 1 (0.2%) |
| 3.Most pulmonary nodules are benign, and there is no need for follow-up or re-examination. | 54 (10.8%) | 60 (12%) | 122 (24.4%) | 227 (45.4%) | 37 (7.4%) |
| 4.If a doctor recommends surgery, I am willing to accept it. | 89 (17.8%) | 199 (39.8%) | 138 (27.6%) | 63 (12.6%) | 11 (2.2%) |
| 5.I believe pulmonary nodules can be controlled by changing lifestyle habits. | 86 (17.2%) | 238 (47.6%) | 115 (23%) | 53 (10.6%) | 8 (1.6%) |
| 6.Pulmonary nodules are likely inflammatory, so taking antibiotics on my own is sufficient. | 50 (10%) | 61 (12.2%) | 169 (33.8%) | 195 (39%) | 25 (5%) |
| 7.I often feel anxious after being diagnosed with pulmonary nodules. | 74 (14.8%) | 113 (22.6%) | 134 (26.8%) | 152 (30.4%) | 27 (5.4%) |
| 8.I am concerned about exposure to X-ray radiation during screening or follow-up. | 70 (14%) | 116 (23.2%) | 136 (27.2%) | 155 (31%) | 23 (4.6%) |
| 9.Smoking is a risk factor for pulmonary nodules, so I am willing to try to quit smoking | 162 (32.4%) | 251 (50.2%) | 57 (11.4%) | 22 (4.4%) | 8 (1.6%) |

**Supplementary Table 4. Distribution of practice dimension responses.**

| **Practice** | **Always** | **Often** | **Sometimes** | **Rarely** | **Never** |
| --- | --- | --- | --- | --- | --- |
| 1. You can follow medical advice and undergo regular check-ups. | 187 (37.4%) | 195 (39%) | 95 (19%) | 21 (4.2%) | 2 (0.4%) |
| 2. How often do you encounter the following risk factors in daily life? | 46 (9.2%) | 126 (25.2%) | 160 (32%) | 151 (30.2%) | 17 (3.4%) |
| 2.1Excessive oil smoke during cooking. | 12 (2.4%) | 21 (4.2%) | 51 (10.2%) | 191 (38.2%) | 225 (45%) |
| 2.2Exposure to asbestos, dust, etc. | 60 (12%) | 93 (18.6%) | 113 (22.6%) | 174 (34.8%) | 60 (12%) |
| 2.3Smoking/Secondhand smoke. | 32 (6.4%) | 63 (12.6%) | 153 (30.6%) | 192 (38.4%) | 60 (12%) |
| 2.4Air pollution. | 54 (10.8%) | 112 (22.4%) | 151 (30.2%) | 147 (29.4%) | 36 (7.2%) |
| 3. You actively seek information about pulmonary nodules. | 35 (7%) | 87 (17.4%) | 162 (32.4%) | 165 (33%) | 51 (10.2%) |
| 4. Discovering a pulmonary nodule causes significant psychological stress for you. | 162 (32.4%) | 209 (41.8%) | 96 (19.2%) | 27 (5.4%) | 6 (1.2%) |
| 5. If a pulmonary nodule is detected, how often would you consider the following further examinations or treatments? | 29 (5.8%) | 63 (12.6%) | 170 (34%) | 133 (26.6%) | 105 (21%) |
| 5.1Regular follow-ups. | 13 (2.6%) | 23 (4.6%) | 69 (13.8%) | 172 (34.4%) | 223 (44.6%) |
| 5.2Taking antibiotics. | 17 (3.4%) | 21 (4.2%) | 76 (15.2%) | 139 (27.8%) | 247 (49.4%) |
| 5.3Needle biopsy. | 17 (3.4%) | 25 (5%) | 88 (17.6%) | 148 (29.6%) | 222 (44.4%) |
| 5.4 Radiofrequency or microwave ablation. | 21 (4.2%) | 44 (8.8%) | 75 (15%) | 112 (22.4%) | 248 (49.6%) |
| 5.5Surgical resection. | 187 (37.4%) | 195 (39%) | 95 (19%) | 21 (4.2%) | 2 (0.4%) |
| 6. If the psychological stress is overwhelming, you would strongly request the lesion be treated, even if the doctor does not recommend it. | 46 (9.2%) | 126 (25.2%) | 160 (32%) | 151 (30.2%) | 17 (3.4%) |
